# Hypomagnesemia Induced by Long-Term Treatment with Proton-Pump Inhibitors

**DOI:** 10.1155/2015/951768

**Published:** 2015-05-04

**Authors:** Simone Janett, Pietro Camozzi, Gabriëlla G. A. M. Peeters, Sebastiano A. G. Lava, Giacomo D. Simonetti, Barbara Goeggel Simonetti, Mario G. Bianchetti, Gregorio P. Milani

**Affiliations:** ^1^Pediatric Department of Southern Switzerland, Ospedale San Giovanni, 6500 Bellinzona, Switzerland; ^2^Pediatric Nephrology, Children's Hospital and University, 3010 Bern, Switzerland; ^3^Pediatric Neurology, Children's Hospital and University, 3010 Bern, Switzerland; ^4^Pediatric Emergency Department, Foundation IRCCS Ca' Granda Ospedale Maggiore Policlinico, 20122 Milan, Italy

## Abstract

In 2006, hypomagnesemia was first described as a complication of proton-pump inhibitors. To address this issue, we systematically reviewed the literature. Hypomagnesemia, mostly associated with hypocalcemic hypoparathyroidism and hypokalemia, was reported in 64 individuals on long-term proton-pump inhibitors. Hypomagnesemia recurred following replacement of one proton-pump inhibitor with another but not with a histamine type-2 receptor antagonist. The association between proton-pump inhibitors and magnesium metabolism was addressed in 14 case-control, cross-sectional studies. An association was found in 11 of them: 6 reports found that the use of proton-pump inhibitors is associated per se with a tendency towards hypomagnesemia, 2 found that this tendency is more pronounced in patients concurrently treated with diuretics, carboplatin, or cisplatin, and 2 found a relevant tendency to hypomagnesemia in patients with poor renal function. Finally, findings likely reflecting decreased intestinal magnesium uptake were observed on treatment with proton-pump inhibitors. Three studies did not disclose any relationship between magnesium metabolism and treatment with histamine type-2 receptor antagonists. In conclusion, proton-pump inhibitors may cause hypomagnesemia. In these cases, switching to a histamine type-2 receptor antagonist is advised.

## 1. Introduction

First introduced in the late 1980s, proton-pump inhibitors are widely used for the management of conditions related to gastric acid secretion such as duodenal and gastric ulcer, reflux esophagitis, and gastroesophageal reflux disease [1].

The cases of two patients developing severe hypomagnesemia along with hypocalcemia and hypokalemia while being on long-term treatment with a proton-pump inhibitor and resolution after withdrawal were first described in 2006 [2]. Following the initial recognition, the association has been subsequently confirmed in various reports [3]. We systematically reviewed and analyzed the available literature. Our aims were to describe in detail this electrolyte abnormality, to address the underlying mechanisms, and to warn physicians about its occurrence.

## 2. Methods

Between May and October 2014, two of the authors (Simone Janett, Pietro Camozzi) independently conducted a computer-based research of the terms “proton-pump inhibitor[s],” “dexlansoprazole,” “omeprazole,” “esomeprazole,” “lansoprazole,” “pantoprazole,” “rabeprazole,” and “hypomagnes[a]emia” or “magnesium” in the U.S. National Library of Medicine database and in the Web-based Google search engine. For this purpose, we used the principles established by the UK Economic and Social Research Council guidance on the conduct of narrative synthesis and on the Preferred Reporting Items for Systematic Reviews and Meta-Analyses statement. Reports available as an article or as a letter in Dutch, English, French, German, Italian, Portuguese, or Spanish were retained for the analysis. To ensure that the search included all published cases, cross-citation screening was manually performed in the references of the included articles. Poorly documented case reports and cases with electrolyte abnormalities secondary to proton-pump inhibitor associated kidney injury were not included [4].

From each individually described patient with hypomagnesemia (<0.75 mmol/L) on proton-pump inhibitor treatment, we made attempts to obtain the following data: gender; age; details and duration of medication; concurrent conditions or management with drugs inducing hypomagnesemia; and laboratory findings regarding magnesium (including changes after withdrawal and rechallenge with a different proton-pump inhibitor or with a histamine type-2 receptor antagonist), calcium, parathyroid hormone and potassium blood levels, and urine magnesium excretion. From case-control, cross-sectional studies, we collected the following information: study setting; year of publication; origin of the report; number of patients and details; magnesium level; comorbidities and concurrent medications. Finally, we reviewed the United States Food and Drug Administration data on hypomagnesemia as adverse event reported during the use of proton-pump inhibitors.

Numerical data are presented as median and interquartile range and categorical data as relative frequency. Linear regressions with the rank correlation coefficient *r*_*s*_ were performed for analysis. Significance was assumed when *P* < 0.05.

## 3. Results

### 3.1. Search Results

**Figure 1:**
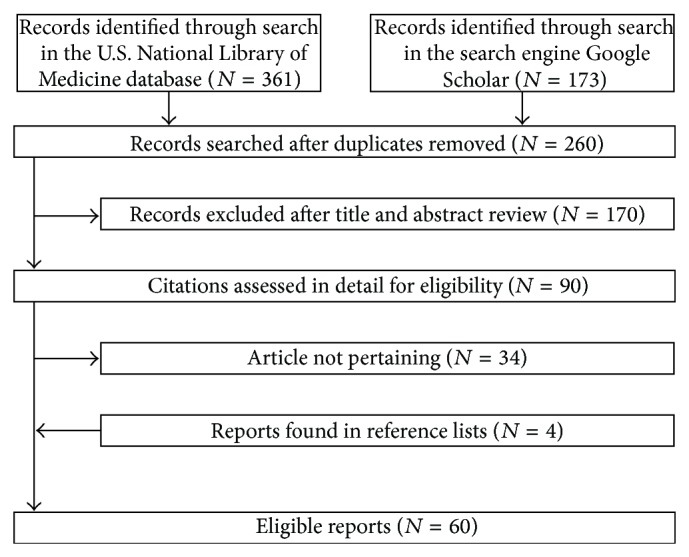
Flowchart of the literature search process. Five of the 60 eligible reports had been identified exclusively from the Web-based search engine Google Scholar.

The initial search revealed 534 publications, of which 260 remained after excluding duplicates (Figure 1). Ninety were reviewed in detail and 56 retained for the final analysis. Four pertinent reports were found in the references of the mentioned reports. Hence, in the final analysis [2, 5–63], we included a total of 60 reports (48 in English, 6 in Spanish, 3 in French, 1 in Dutch, 1 in German, and 1 in Italian) from the United Kingdom (*N* = 12), the United States of America (*N* = 9), Spain (*N* = 7), the Netherlands (*N* = 4), Brazil (*N* = 3), France (*N* = 3), Italy (*N* = 3), Switzerland (*N* = 3), Australia (*N* = 2), Belgium (*N* = 2), Canada (*N* = 2), Germany (*N* = 2), Korea (*N* = 2), Argentina (*N* = 1), Greece (*N* = 1), Israel (*N* = 1), Japan (*N* = 1), New Zealand (*N* = 1), and Turkey (*N* = 1).

They were 45 reports detailing individual cases [2, 19–62], 14 case-control, cross-sectional studies [5–18] and one report based on the United States Food and Drug Administration data [63]. The case of a Spanish patient reported twice in the literature was considered only once [33, 34].

### 3.2. Individual Cases

**Figure 2:**
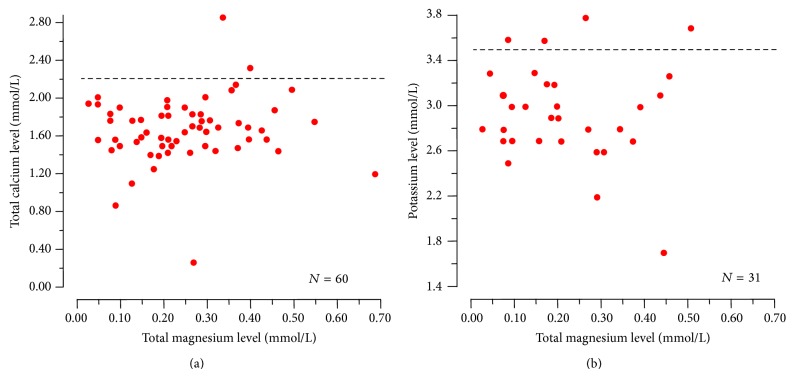
Relationship between circulating magnesium and total calcium (left panel) or potassium (right panel) in patients with proton-pump inhibitor associated hypomagnesemia. None of the correlations was found to be significant. The horizontal dotted lines denote the threshold level of hypocalcemia (2.20 mmol/L) and hypokalemia (3.5 mmol/L), respectively.

The 45 reports detailing cases of proton-pump inhibitor associated hypomagnesemia included a total of 64 individual cases. The characteristics of the patients (30 male and 34 female subjects) appear in Table 1. The age of 53 (83%) of the 64 patients ranged between 50 and 80 years. Hypomagnesemia was rather severe: it ranged between 0.03 and 0.71, with median 0.21 mmol/L and was ≤0.50 mmol/L in 62 (97%) cases. The duration of treatment with a proton-pump inhibitor was one year or more in all cases with the exception of a newborn baby with gastroesophageal reflux on lansoprazole, and 3 years or more in 75% of the cases. When measured, hypomagnesemia was always accompanied by hypomagnesiuria. Moreover, it was very often accompanied by blood calcium level <2.20 mmol/L (97%), low or normal parathyroid hormone levels (95%), and blood potassium level <3.50 mmol/L (87%). Linear regression analysis did not show an association between the degree of hypomagnesemia with the degree of hypocalcemia or hypokalemia (Figure 2). Further possible causes of hypomagnesemia were observed in at least 21 cases: treatment with either thiazide (*N* = 7) or loop diuretic (*N* = 6), alcohol abuse (*N* = 5), poor renal function (*N* = 2), and small bowel resection (*N* = 1).

The following proton-pump inhibitors were used in the 64 patients: omeprazole, esomeprazole, the S-isomer of omeprazole, or both (*N* = 52); pantoprazole (*N* = 6); lansoprazole (*N* = 3); omeprazole and lansoprazole (*N* = 1); rabeprazole (*N* = 2). No cases of hypomagnesemia were reported on dexlansoprazole. Hypomagnesemia resolved after discontinuing the proton-pump inhibitor but recurred following replacement with another proton-pump inhibitor in at least 13 cases. In these cases, the initially used proton-pump inhibitor was replaced as follows: with omeprazole or esomeprazole (*N* = 4); with lansoprazole (*N* = 3); with pantoprazole (*N* = 2); with esomeprazole and subsequently pantoprazole (*N* = 2); and with pantoprazole and subsequently lansoprazole (*N* = 2). In contrast, hypomagnesemia did not recur in 32 patients following switching to a histamine type-2 receptor antagonist: ranitidine (*N* = 29), famotidine (*N* = 2), and cimetidine (*N* = 1).

### 3.3. Case-Control, Cross-Sectional Studies

The association between proton-pump inhibitors and metabolism of magnesium was addressed in 14 case-control, cross-sectional studies, as given in Table 2. An association was found uniquely in 10 of them. Six reports found that the use of proton-pump inhibitors is associated per se with a tendency towards hypomagnesemia [8, 10, 11, 13, 14, 18]. Two reports found that the tendency towards hypomagnesemia is more pronounced in patients concurrently treated with either diuretic [13, 18] or carboplatin and cisplatin [11]. Danziger et al. [7] found that hypomagnesemia occurs exclusively in patients concurrently taking diuretics. Furthermore, two reports found a relevant tendency to hypomagnesemia in patients with poor renal function treated with proton-pump inhibitors [5, 15].

William et al. [17] found an association between the use of proton-pump inhibitors and decreased urinary magnesium excretion (a finding that likely reflects a decreased intestinal magnesium uptake).

Finally, three of the aforementioned studies did not find any relationship between magnesium and histamine type-2 receptor antagonists [7, 14, 18].

### 3.4. United States Food and Drug Administration Data

The reports submitted to the Adverse Event Reporting System of the United States Food and Drug Administration were recently examined to address the association between use of proton-pump inhibitors and hypomagnesemia [63]. Between 1997 and 2012, 66,102 subjects were identified as experiencing at least one adverse effect while taking a proton-pump inhibitor. Among the mentioned subjects, 693 (=1.0%) were reported to have hypomagnesemia. Subjects aged ≥65 years were at increased risk of hypomagnesemia (*P* < 0.001). Finally, there was a strong (*P* < 0.001) association between occurrence of hypomagnesemia and that of hypocalcemia or hypokalemia. Two thirds (*N* = 461) of the hypomagnesemia cases occurred in subjects treated with omeprazole or esomeprazole, the most frequently prescribed proton-pump inhibitors.

## 4. Discussion

This work brings together the most recent literature and some reports identified exclusively from the search engine Google Scholar. Its results, acquired both in individual case reports and in case-control, cross-sectional studies, support the hypothesis that management with proton-pump inhibitors may cause hypomagnesemia along with hypomagnesiuria, hypocalcemia, and hypokalemia (see the following list). This tendency is more pronounced in patients concomitantly treated with cisplatin, carboplatin, and especially diuretics and in those with poor renal function.

*Clinical and Biochemical Characteristics of Proton-Pump Inhibitor Associated Hypomagnesemia.* Consider the following.

- Age mostly ≥50 years, duration of treatment ≥1 year, and no emblematical patient profile unique for this electrolyte abnormality, being more frequent in patients concurrently managed with other factors that may lower magnesemia (e.g., management with thiazides and loop diuretics, alcohol abuse) and in those with poor renal function.
- Hypomagnesemia often accompanied by hypocalcemic hypoparathyroidism and hypokalemia.
- Hypomagnesemia resolution soon after discontinuing the proton-pump inhibitor, recurring upon readministration or after replacement of one proton-pump inhibitor with another (=class effect).
- Hypomagnesemia not occurring with histamine type-2 receptor antagonists (e.g., ranitidine).

The concurrent demonstration of both magnesium deficiency and hypomagnesiuria suggests that impaired intestinal magnesium absorption is the culprit. This electrolyte abnormality recovers within 4 days after discontinuing the drug; it recurs upon readministration or after replacement with another proton-pump inhibitor [64], but it does not develop with other acid suppressants such as histamine type-2 receptor antagonists. Hypomagnesemia was mostly accompanied by hypocalcemia and normal or low parathyroid hormone levels, confirming the existence of a state of secondary hypoparathyroidism in the most severely hypomagnesemic patients [65]. A further common laboratory finding was hypokalemia, a recognized consequence of hypomagnesemia [65].

In the few individual cases available for such an analysis, no significant correlation was noted between circulating magnesium and hypocalcemia or hypokalemia. At least two factors might account for this unexpected observation. First, there are sometimes differences among laboratories with respect to total magnesium determination in blood [65]. Furthermore, circulating magnesium exists in the ionized, biologically active state and in the undissociated form, either bound to albumin or complexed to anions such as bicarbonate, citrate, and phosphate [65].

At least three limitations of our study should be mentioned. First, the analysis results rather from the small number of reported subjects affected by proton-pump inhibitor associated hypomagnesemia. Second, because of the scant information available on the clinical presentation of magnesium deficiency, the present study does not address this issue. Third, the underlying mechanisms require further investigations.

Recognition and management of hypomagnesemia secondary to long-term treatment with proton-pump inhibitors mainly rest on two pillars: first, blood magnesium monitoring at least in patients with symptoms or signs consistent with magnesium deficiency, in those concurrently treated with other agents that may lower magnesium level, and in those with poor renal function; second, in patients with hypomagnesemia, switching to a histamine type-2 receptor antagonist may be attempted. Further studies are required to identify whether magnesium supplements or sucralfate might be prescribed in subjects with proton-pump inhibitor associated hypomagnesemia.

## Figures and Tables

**Table 1:** Clinical and laboratory findings in 64 patients with hypomagnesemia (<0.75 mmol/L) while being on long-term management with a proton-pump inhibitor. Numerical data are presented as median and interquartile range (which extends from the value at centile 25 to that at centile 75 and includes half of the data points) and categorical data as relative frequency.

Age, years	66 [59–73]
Gender, ♂/♀	30/34
Circulating magnesium level, mmol/L	0.24 [0.15–0.30]
Duration of treatment with proton-pump inhibitor, years	4 [3–10]
Urinary magnesium excretion, low/normal	42/0
Circulating calcium level, low/normal	58/2
Parathyroid hormone level^‡^, low-normal/high	42/2
Circulating potassium level, low^∗^/normal	27/4

^‡^Assessed exclusively in subjects with total calcium level <2.20 mmol/L. ^∗^<3.5 mmol/L.

**Table 2:** Case-control, cross-sectional studies addressing the potential of proton-pump inhibitors to modulate the metabolism of magnesium. Reports disclosing a significant relationship between use of these drugs and metabolism of magnesium are bold.

Reference	Country	Patients	Results
**Alhosaini et al.** [5]	**USA**	**62 hemodialysis patients**	**Use of proton-pump inhibitors was associated** (**P** < 0.05) **with hypomagnesemia**

Biyik et al. [6]	Turkey	238 outpatients	Magnesemia was similar in users of proton-pump inhibitors (*N* = 154) and nonusers (*N* = 84)

**Danziger et al.** [7]	**USA**	**11,490** ** patients admitted to an intensive care unit **	**Hypomagnesemia was disclosed exclusively** (**P** < 0.01) **in patients concurrently treated with both proton-pump inhibitors and diuretics. Use of histamine type-2 receptor antagonists** (**with or without diuretics**) **was not associated with hypomagnesemia**

**El-Charabaty et al.** [8]	**USA**	**262 intensive care patients with cardiac arrhythmias**	**Use of proton-pump inhibitors prior to admission was associated with tendency to hypomagnesemia** (**r** = 0.82)

Faulhaber et al. [9]	Brazil	151 internal medicine patients	No cases of hypomagnesemia were detected on treatment with proton-pump inhibitors

**Gau et al.** [10]	**USA**	**487 inpatients**	**Use of proton-pump inhibitors was associated with lower magnesemia** (**P** < 0.005) **and with a 2.5-fold increased risk of hypomagnesemia**

**Kim et al.** [11]	**Korea**	**1356 patients**	**Magnesemia was lower** (**P** < 0.0001) **in users of proton-pump inhibitors** (**N** = 112) **than in nonusers** (**N** = **1,244**). **Concurrent treatment with cisplatin** (**P** < 0.05) **or carboplatin** (**P** < 0.01) **further exacerbated hypomagnesemia**

Koulouridis et al. [12]	USA	804 well-matched inpatients	Use of proton-pump inhibitors prior to admission was not associated with hypomagnesemia

**Lindner et al.** [13]	**Switzerland**	**5,118 emergency department patients**	**Hypomagnesemia was significantly** (**P** < 0.0001) **associated with the use of proton-pump inhibitor or both proton-pump inhibitors and diuretics**

**Markovits et al.** [14]	**Israel**	**95,205 outpatients**	**Users of proton-pump inhibitors** (**N** = **22,458**) **presented frequently with hypomagnesemia** (**P** < 0.005). **Use of histamine type-2 receptor antagonists was not associated with hypomagnesemia**

**Sumukadas et al.** [15]	**United Kingdom**	**196 elderly unit patients**	**Magnesemia was lower** (**P** < 0.05) **in users of proton-pump inhibitors with poor renal function**

Van Ende et al. [16]	Belgium	512 renal transplant recipients	Use of proton-pump inhibitors was not a predictor of hypomagnesemia

**William et al.** [17]	**USA**	**278 outpatients**	**Magnesiuria was reduced** (**P** < 0.02) **in users of proton-pump inhibitors**. **Diuretics did not modulate the effect of proton-pump inhibitors on magnesiuria**

**Zipursky et al.** [18]	**Canada**	**1,830 inpatients**	**Patients admitted with hypomagnesemia were frequently** (**odds ratio** = **1.43**) **on proton-pump inhibitors**. **Diuretics further exacerbated the tendency to hypomagnesemia** (**odds ratio** = **1.73**). **Use of histamine type-2 receptor antagonists was not associated with hypomagnesemia**
